# Factors associated with non-use of modern contraceptive methods in the school-going adolescent’s girls and young women in Guinea

**DOI:** 10.4102/jphia.v17i1.1670

**Published:** 2026-06-30

**Authors:** Sidikiba Sidibé, Djiba Diakité, Almamy A. Touré, Aboubacar S. Magassouba, Hadja F. Camara, Abdoulaye Sow, Mory 1 Kourouma, Facely Camara, Ansoumane Sidibé, Alexandre Delamou

**Affiliations:** 1 African Centre of Excellence for the Prevention and Control of Transmissible Diseases, Gamal Abdel Nasser University, Conakry, Guinea; 2 Department of Public Health, Faculty of Sciences and Health Techniques, Gamal Abdel Nasser University, Conakry, Guinea; 3 Maferinyah National Centre for Training and Research in Rural Health, Forécariyah, Guinea; 4 Faculty of Sciences and Health Techniques, Gamal Abdel Nasser University, Conakry, Guinea

**Keywords:** unmarried youth, young women, sexually active, modern contraception, schools, Guinea

## Abstract

**Background:**

Non-use of modern contraceptive methods among adolescents and young women remains prevalent in Africa.

**Aim:**

This study analysed factors associated with the non-use of modern contraceptives among unmarried, sexually active school-going adolescent girls and young women in Guinea.

**Setting:**

The study was conducted among sexually active school-going adolescent girls and young women in Guinea.

**Methods:**

A cross-sectional survey using a three-stage sampling approach was conducted from December 2020 to March 2021 among female students (13–24 years) in secondary schools. Multivariable logistic regression identified factors associated with non-use, reporting adjusted odds ratios (AOR) with 95% confidence intervals (CI).

**Results:**

The prevalence of non-use was 48.2% (95% CI: 46.3–50.2). Significantly higher odds of non-use were associated with: college-grade level (AOR: 1.67, CI: 1.29–2.17), private schooling (AOR: 1.69, CI: 1.29–2.21), being Muslim (AOR: 4.23, CI: 2.27–7.88) or Christian (AOR: 3.01, CI: 1.62–5.62), no contraceptive knowledge (AOR: 11.95, CI: 7.08–20.1), wanting a birth within 2 years (AOR: 2.51, CI: 1.98–3.17), partner opposition compliance (AOR: 2.05, CI: 1.53–2.74), family planning needs (AOR: 1.68, CI: 1.31–2.15), and poor contraceptive access (AOR: 3.87, CI: 2.97–5.04).

**Conclusion:**

Modern contraceptive non-use is a stark reality among unmarried school-going youth in Guinea. Implementing robust school-based family planning education programmes is crucial to improve knowledge and uptake.

**Contribution:**

This study guides school sexual health policies, transforming a taboo issue into an opportunity for informed public health action.

## Introduction

Each year between 2015 and 2019, 121 million undesirable pregnancies were recorded worldwide, 61% of which resulted in abortion.^1^ In 2021, out of 1.9 billion women of reproductive age, a substantial number (874 million) were using modern contraceptives, while 164 million women were unable to access the contraception they required.^2^ The percentage of women in their reproductive years who utilise modern family planning methods is tracked as part of Sustainable Development Goal indicator 3.7.1.^3^

Contraceptive use has been studied in various populations and contexts. A systematic review and meta-analysis covering the period 1999–2018 revealed that 57% of unmarried women used contraceptive methods.^4^ In a study of sexually active teenage girls in school in nine sub-Saharan African countries, 34.4% had not used contraceptives during their last act of sex.^5^ In one city in Ethiopia, 41.5% of women of reproductive age had not adopted modern contraception.^6^ A national school survey conducted in Mozambique in 2015 showed that 42% had not used contraception the last time they had sexual intercourse.^7^

Between 2005 and 2015, a systematic review conducted in sub-Saharan Africa identified various factors that hindered contraceptive use, including perceptions of adverse effects, lack of spousal approval and social norms related to fertility. On the contrary, positive factors included education, employment and communication with a male partner.^8^ Additional factors have been identified that impact the use of methods of contraception, including the woman’s age, early sexual debut, having several sexual partners, lack of parental support and lack of knowledge.^5,9^ These findings were also shared by other studies.^6,10^

Despite high maternal mortality rates in Guinea, contraceptive use remains low among women of reproductive age. The results from the 2018 Demographic and Health Surveys indicate a prevalence rate of 11.0% among women aged 15–49 years.^11^ However, studies on contraception in Guinea focus mainly on adolescents and young women, among whom the use of modern methods has increased slightly, from 8.4% to 12.8% between 1999 and 2018.^12^ In terms of contraceptive needs, 22.6% of adolescents and young women had unsatisfied contraceptive needs.^13^ In Guinea, the main factors determining contraceptive use included fear of adverse effects, approval from the male partner, social, cultural and religious norms, the competence of health care providers, as well as household wealth.^12,14,15^ Available data indicate a prevalence of early sexual intercourse of 32.7%^16^ and pregnancies of 13.1%^17^ among school-going adolescents. Despite these findings, the utilisation of modern contraceptive methods remains low, as shown by a study in rural areas revealing a prevalence of 24.4% among school-going girls.^15^ Most existing research has been limited to local contexts and does not provide a picture of the situation at the national level, more specifically among sexually active, non-married teenage girls and young women in school contexts, who constitute a population at risk. It is within this context that the present study was carried out to identify the factors associated with the non-use of modern contraceptive methods among sexually active, unmarried, school-going adolescent girls and young women in Guinea.

## Research methods and design

### Study design

This study was conducted in 2021 in schools (middle schools and high schools) in Guinea as part of a doctoral research project.

### Setting

The research was conducted in public and private schools (middle and high schools) in six regions of Guinea (Conakry, Faranah, Kankan, Kindia, Labé and N’zérékoré) and included all students present in class at the time of the survey.

### Study population and sampling strategy

The study population consisted of adolescent girls and young women enrolled in secondary schools in Guinea. The World Health Organization defines adolescents as individuals aged 10–19 years, while young people are defined as those aged 15–24 years.^18^ Sampling in each region was carried out in three stages: firstly, schools were selected on a targeted basis; secondly, classes were chosen by grade level, with priority given to classes with the most students; thirdly, students were selected at random using a systematic approach.

### Data collection and variables

**FIGURE 1:**
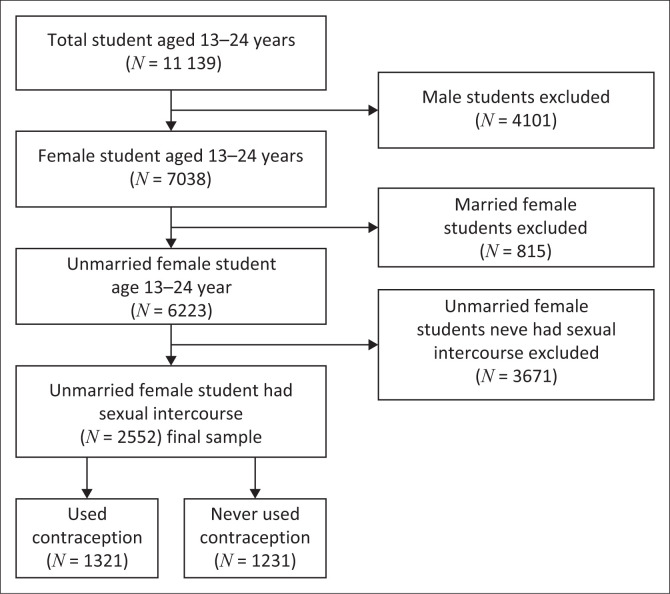
Sample selection flow chart.

Figure 1 illustrates the sample selection criteria for this study, which initially included students aged 13–24 years. The sample size was derived from the initial population of students aged 13–24 years (*N* = 11 139) through a successive exclusion process based on predefined criteria. Initially, boys were excluded, followed by married females and then unmarried females who had never had sexual intercourse. Ultimately, only sexually active unmarried females were retained, forming the final study sample. The final sample comprised 2552 unmarried sexually active students.

The outcome variable in this study was the lack of use of modern contraceptive methods (modern methods of contraception include intrauterine devices, implants or Norplants, injectable contraceptives, oral contraceptives, condoms and sterilisation) among sexually active, non-married, school-going adolescents and young women. Those who had never used a modern contraceptive method were coded ‘1’. Those who used at least one modern contraceptive method were coded ‘0’.

The study’s independent variables included age, educational level, school type, religion and region. Additionally, adolescents’ girls were questioned about their knowledge of contraceptive methods, having heard of contraception, wanting a child within the next 2 years, thinking that men should authorise the use of contraceptive methods, opinion on contraception, having a sexual partner at the moment of the interview, being willing to use a contraceptive method even if the partner opposes it, having contraceptive needs and ease of access to contraceptive methods. Age was recoded into two classes (13–19 years and 20–24 years). Education level was recorded into middle school (Grades 7–10) and high school (Grades 11–13) and school type into public and private. Knowledge was assessed using three indicators: knowledge of at least one contraceptive method, knowledge of a benefit of family planning and knowledge of a family planning service delivery location, each coded as ‘1’ for a positive response. A composite score ranging from 0 to 3 was then calculated by summing these items. Based on this score, participants were categorised into three levels of knowledge: none (0 points), moderate (1–2 points) and high (3 points). Students who had not used any contraceptive methods were asked about the reasons for non-use.

### Data analysis

The data were imported into Stata software, version 16 (StataCorp LLC, College Station, TX, United States [US]) for analysis, and their completeness and coherence were carefully checked. Descriptive statistics were calculated, including means, standard deviations, frequencies and percentages. To assess potential multicollinearity among the explanatory variables, the variance inflation factor was applied. Associations between categorical variables were examined using Pearson’s chi-square test. Explanatory variables with a *p*-value < 0.25 in the bivariate logistic regression were selected and subsequently included in the multivariable analysis. Multivariable logistic regression was performed to explore the relationship between independent variables and the outcome. Each variable was adjusted for the presence of other variables in the model. Adjusted odds ratios (AOR) with 95% confidence intervals (CIs) were reported, and a *p*-value less than 0.05 was considered statistically significant.

### Ethical considerations

Ethical clearance to conduct this study was obtained from the National Ethics Committee for Health Research (CNERS) (No. 045/CNERS/19). Ethical authorisation was not necessary, as the primary study was obtained after obtaining ethical authorisation from the National Ethics Committee of Guinea. The authors obtained permission to use this dataset.

## Results

### Characteristics of the participants

The 13–19 years age group was the most represented (74.4%), around 77% studied in public schools, 83.6% had heard of contraception and 46.6% had a high knowledge of contraceptive methods. Only 16.6% thought that men should authorise the use of contraceptive methods, 23% disapproved of contraceptive methods, 86% had a sexual partner at the moment of the interview, 76.8% were prepared to use a contraceptive method even if their partner objected and almost 61% had easy access to contraceptive methods (Table 1).

**TABLE 1:** Sociodemographic characteristics of adolescent girls and young women attending school in Guinea, 2021 (*N* = 2552).

Variables	Categories	*n*	%
Age (years)	13–19	1899	74.41
	20–24	653	25.59
Level of education	College	1413	55.37
	High school	1139	44.63
Type of school	Public	1962	76.88
	Private	590	23.12
Religion	Muslim	1511	59.21
	Christian	895	35.07
	Others†	146	5.72
Region	Conakry	1097	42.99
	Faranah	227	8.89
	Kankan	272	10.66
	Kindia	171	6.70
	Labé	59	2.31
	Nzérékoré	726	28.45
Having heard about contraception	No	418	16.38
	Yes	2134	83.62
Knowledge of the benefits of contraception	No	758	29.70
	Yes	1794	70.30
Knowledge of where family planning services are dispensed	No	874	34.25
	Yes	1678	65.75
Level of knowledge about family planning	None	379	14.85
	Moderate	983	38.52
	High	1190	46.63
Want a birth in the next 2 years	No	1373	53.80
	Yes	1179	46.20
Thinking that men should authorise the use of contraceptive methods	No	2129	83.42
	Yes	423	16.58
Perception of contraceptive methods	Disapprove	501	23.12
	Approve	1666	76.88
Having a sexual partner	No	333	13.65
	Yes	2106	86.35
Willing to use contraceptive methods even if partner is opposed	No	539	23.23
	Yes	1781	76.77
Have family planning needs	No	1731	67.83
	Yes	821	32.17
Easy access to contraceptive methods	No	996	39.03
	Yes	1556	60.97

† , Traditionalist or no religion.

### Prevalence of non-use of modern contraceptive methods among adolescents and young women attending school

The prevalence of non-use of modern contraceptive methods among participants was 48.2% (46.3–50.2). Middle school students (50.5%), private school students (59%), Muslims (56%), students who had no knowledge of contraceptive methods (90.5%), those who wanted a birth within the next 2 years (61%), those who thought that men should authorise the use of contraceptive methods (69.3%), those who disapproved of the use of contraceptive methods (62.5%), those who are not having a sexual partner (54.7%), students who were not prepared to use contraceptive methods if their partner objected (70.5%), those who had family planning needs (51.7%) and those who did not have easy access to contraceptive methods when needed (78.2%) showed higher non-use of modern contraceptive methods within their respective categories. No statistically significant variation was found among the different age categories (Table 2).

**TABLE 2:** Prevalence of non-use of modern contraceptive methods among adolescents and young women attending school in Guinea, 2021.

Variables	Categories	No (*n* = 1321)		Yes (*n* = 1231)		*p*-value
		*n*	%	*n*	%	
**Age (years)**	-	-	-	-	-	0.085
	13–19	964	50.76	935	49.24	-
	20–24	357	54.67	296	45.33	-
**Level of education**	-	-	-	-	-	0.012
	College	700	49.54	713	50.46	-
	High school	621	54.52	518	45.48	-
**Type of school**	-	-	-	-	-	< 0.001
	Public	1080	55.05	882	44.95	-
	Private	241	40.85	349	59.15	-
**Religion**	-	-	-	-	-	< 0.001
	Muslim	662	43.81	849	56.19	-
	Christian	537	60.00	358	40.00	-
	Others†	122	83.56	24	16.44	-
**Region**	-	-	-	-	-	< 0.001
	Conakry	609	55.52	488	44.48	-
	Faranah	40	17.62	187	82.38	-
	Kankan	66	24.26	206	75.74	-
	Kindia	60	35.09	111	64.91	-
	Labé	1	1.69	58	98.31	-
	Nzérékoré	545	75.07	181	24.93	-
**Level of knowledge about family planning**	-	-	-	-	-	< 0.001
	None	36	9.50	343	90.50	-
	Moderate	354	36.01	629	63.99	-
	High	931	78.24	259	21.76	-
**Want a birth in the next 2 years**	-	-	-	-	-	< 0.001
	No	785	66.58	394	33.42	-
	Yes	536	39.04	837	60.96	
**Thinking that men should authorise the use of contraceptive methods**	-	-	-	-	-	< 0.001
	No	1191	55.94	938	44.06	-
	Yes	130	30.73	293	69.27	-
**Perception of contraceptive methods**	-	-	-	-	-	< 0.001
	Disapprove	188	37.52	313	62.48	-
	Approve	1066	63.99	600	36.01	-
**Having a sexual partner**	-	-	-	-	-	0.003
	No	151	45.35	182	54.65	-
	Yes	1141	54.18	965	45.82	-
**Willing to use contraceptive methods even if partner is opposed**	-	-	-	-	-	< 0.001
	No	159	29.50	380	70.50	-
	Yes	1108	62.21	673	37.79	-
**Have family planning needs**	-	-	-	-	-	< 0.001
	No	836	48.30	895	51.70	-
	Yes	485	59.07	336	40.93	-
**Easy access to contraceptive methods**	-	-	-	-	-	< 0.001
	No	217	21.79	779	78.21	-
	Yes	1104	70.95	452	29.05	-

Note: Prevalence of non-use of modern contraceptive methods (95% CI) = 48.24% (46.30–50.18).

† , Traditionalist or no religion.

### Reasons why contraceptive methods are not utilised

The main reasons given by students (*n* = 1231) for not using contraceptive methods were failure of previous contraception and partner’s opposition (3%), negative beliefs or rumours (4%), high cost and difficulty of access (5%), reduced sexual satisfaction (10%), lack of information (13%), fear of parents (25%), religious barrier (30%) and fear of side effects (59%). Other reasons cited were difficulty of access, negative perceptions or beliefs about contraception, opposition from a partner and failure of previous use.

### Factors associated with non-use of modern contraceptive methods among adolescents and young women attending school

Several factors were associated with non-use of modern contraceptive methods among school-attending adolescents and young women. Middle school students had higher odds of non-use (AOR: 1.67, 95% CI: 1.29–2.17) than high school students, and students in private schools had higher odds of non-use (AOR: 1.69, 95% CI: 1.29–2.21) than those in public schools. Religious affiliation was also a significant factor, with Muslim students (AOR: 4.23, 95% CI: 2.27–7.88) and Christian students (AOR: 3.01, 95% CI: 1.62–5.62) less likely to use modern contraceptives than students of other religious denominations (traditionalists and no religious denomination). Knowledge of contraceptive methods strongly influenced use. Students with no knowledge (AOR: 11.95, 95% CI: 7.08–20.1) or moderate knowledge (AOR: 4.57, 95% CI: 3.57–5.86) were much less likely to use modern contraceptives than those with high knowledge. Planning a birth within the next 2 years (AOR: 2.51, 95% CI: 1.98–3.17), unwillingness to use contraception if their partner objected (AOR: 2.05, 95% CI: 1.53–2.74) and having unmet family planning needs (AOR: 1.68, 95% CI: 1.31–2.15) were also associated with higher odds of non-use. Limited access to contraceptive methods markedly increased non-use (AOR: 3.87, 95% CI: 2.97–5.04) (Table 3).

**TABLE 3:** Factors associated with non-use of modern contraceptive methods among adolescents and young women attending school in Guinea, 2021.

Variables	Categories	AOR	95% CI	*p*
**Age (years)**	-	-	-	0.487
	13–19	Ref.	-	-
	20–24	1.10	0.84–1.46	-
**Level of education**	-	-	-	< 0.001
	College	1.67	1.29–2.17	-
	High school	Ref.	-	-
**Type of school**	-	-	-	< 0.001
	Public	Ref.	-	-
	Private	1.69	1.29–2.21	-
**Religion**	-	-	-	< 0.001
	Muslim	4.23	2.27–7.88	0.001
	Christian	3.01	1.62–5.62	-
	Others†	Ref.	-	-
**Level of knowledge about family planning**	-	-	-	< 0.001
	None	11.95	7.08–20.1	< 0.001
	Moderate	4.57	3.57–5.86	
	High	Ref.	-	-
**Want a birth in the next 2 years**	-	-	-	< 0.001
	No	Ref.	-	-
	Yes	2.51	1.98–3.17	-
**Thinking that men should authorise the u se of contraceptive methods**	-	-	-	0.220
	No	Ref.	-	-
	Yes	1.24	0.88–1.73	-
**Perception of contraceptive methods**	-	-	-	0.731
	Disapprove	Ref.	-	-
	Approve	1.05	0.78–1.42	-
**Having a sexual partner**	-	-	-	0.228
	No	Ref.	-	-
	Yes	1.25	0.87–1.79	-
**Willing to use contraceptive methods even if partner is opposed**	-	-	-	< 0.001
	No	2.05	1.53–2.74	-
	Yes	Ref.	-	-
**Have family planning needs**	-	-	-	< 0.001
	No	Ref.	-	-
	Yes	1.68	1.31–2.15	-
**Easy access to contraceptive methods**	-	-	-	< 0.001
	No	3.87	2.97–5.04	-
	Yes	Ref.	-	-

AOR, adjusted odds ratio; CI, confidence interval; Ref., reference category.

† , Traditionalist or no religion.

## Discussion

The objective of this study was to identify the factors influencing the use of modern contraceptive methods among sexually active unmarried adolescents and young women attending schools in Guinea. This prevalence of the non-use of modern contraceptives was 48.2%. By comparison, in urban areas of northeastern Mexico, 34.9% of sexually active adolescents reported not using contraception at first sexual intercourse.^9^ In nine sub-Saharan African countries, 34.4% of sexually active adolescents in school had not used a means of contraception during their last sexual intercourse.^5^ In Mozambique, 42% of teenagers attending school had not used any means of contraception during their last sexual intercourse.^7^ These studies were conducted among adolescent girls only, whereas ours included adolescent girls and young women, which could explain this difference. In our study, the primary reasons for not using modern contraceptive methods were reduced sexual satisfaction (10%), lack of information (13%), fear of parents (25%), religious barriers (30%) and fear of side effects (59%). These findings are consistent with previous studies indicating that concerns about health risks and limited availability of contraceptive methods, shame and cultural or religious barriers limit the use of contraceptive methods.^14,19,20,21,22,23^ It is essential to enhance education and understanding of reproductive health to promote the uptake of contemporary contraceptive options.

### Factors influencing the use of modern contraceptive methods

In our study, several factors influenced the use of modern contraceptive methods among unmarried sexually active teenagers and young women, including level of education, type of school, level of knowledge, religion, wanting a baby within the next 2 years and not having easy access to contraceptive methods.

College girls had higher odds of non-use of the modern contraceptive methods compared to high school girls. Several findings have shown that a low level of education is associated with non-use of modern contraceptive methods.^6,24,25^ This could be attributed to the fact that higher education enhances understanding of health-related determinants. Educated women have improved access to health information and greater autonomy to make decisions regarding their own health, including the adoption of contraceptive methods.

Students in private schools were more likely to refrain from using modern contraceptive methods, possibly because many perceive themselves as more independent than those attending public schools.

Low knowledge of contraceptive methods was associated with non-use in our study. Knowledge is a key predisposing factor that encourages positive health behaviours, including the uptake of contraceptive methods. Evidence from multiple studies indicates that insufficient knowledge is a major contributor to women’s non-use of contraceptives.^9,25,26,27,28^ Efforts should be made to increase knowledge and the adoption of contraceptive methods among adolescent girls and young women.

Sexually active Muslim and Christian women were less likely to use modern contraceptives than traditionalists and those with no religion. This result corroborates those of other studies.^29,30^

Religious beliefs can hinder women’s adoption of modern contraceptive methods. In our study, 30% of participants who had never used a modern method reported religion as a barrier. Similarly, a study exploring contraceptive practices among Muslim women in the United States found that 35% of respondents believed that Islam prohibits the use of permanent contraceptive methods.^31^

Adolescent girls and young women planning a pregnancy in the next 2 years were less likely to adopt modern contraceptive methods. Similarly, research in the Kigoma region of Tanzania showed that women aged 15–49 years who wanted to postpone childbirth by at least 2 years were four times more likely to use contraceptives.^32^ A woman’s desire to become pregnant within a short space of time is an obstacle to using a contraceptive method.

In our study, teenage girls and young women who were unwilling to use contraceptive methods if their partner objected were more likely to be non-users. Several studies have shown that women whose partners had an unfavourable opinion of the use of modern contraceptive methods were less likely to use them.^6,8,14^

In our study, adolescents and young women with family planning needs and those without easy access to contraceptive methods were more likely to be non-users. These results corroborate those of the literature.^14,29,32,33^ Easy access to free or low-cost contraceptives is a key determinant of their utilisation.

Our study has some limitations. As a result of its cross-sectional nature, it cannot establish a causal link between non-utilisation of modern contraceptives among sexually active, unmarried adolescent girls and young women and the associated factors. As a result of the cultural sensitivity surrounding sexuality and the use of contraceptive methods, responses could be subject to social desirability bias. In addition, this study was unable to assess the influence of other factors such as parental control, multiple sexual partners and the consumption of psychoactive substances, including tobacco and other drugs. Despite this, the present study of a nationally representative sample provides evidence that could help policymakers implement interventions to increase the prevalence of the adoption of modern contraceptive methods among school students, offering a basis for subsequent studies.

## Conclusion

Nearly half of sexually active unmarried teenagers and young women in schools have never used modern contraceptive methods. The study identified several associated factors as the lack of use of modern contraceptive methods. One important associated factor is the low level of knowledge about family planning. It would therefore be essential to implement educational programmes on family planning to strengthen women’s understanding of modern contraceptive methods. In addition, these programmes should pay particular attention to the evidence-based interventions demonstrated by this research that could help policymakers in the decision-making process regarding the use of modern contraceptive methods in schools.
